# An Extended Nomenclature for Mammalian V-ATPase Subunit Genes and Splice Variants

**DOI:** 10.1371/journal.pone.0009531

**Published:** 2010-03-10

**Authors:** Kevin C. Miranda, Fiona E. Karet, Dennis Brown

**Affiliations:** 1 Program in Membrane Biology and Division of Nephrology, Center for Systems Biology, Massachusetts General Hospital, Boston, Massachusetts, United States of America; 2 Harvard Medical School, Boston, Massachusetts, United States of America; 3 Department of Medical Genetics, University of Cambridge, Cambridge, United Kingdom; Science Commons, United States of America

## Abstract

The vacuolar-type H^+^-ATPase (V-ATPase) is a multisubunit proton pump that is involved in both intra- and extracellular acidification processes throughout the body. Multiple homologs and splice variants of V-ATPase subunits are thought to explain its varied spatial and temporal expression pattern in different cell types. Recently subunit nomenclature was standardized with a total of 22 subunit variants identified. However this standardization did not accommodate the existence of splice variants and is therefore incomplete. Thus, we propose here an extension of subunit nomenclature along with a literature and sequence database scan for additional V-ATPase subunits. An additional 17 variants were pulled from a literature search while 4 uncharacterized potential subunit variants were found in sequence databases. These findings have been integrated with the current V-ATPase knowledge base to create a new V-ATPase subunit catalogue. It is envisioned this catalogue will form a new platform on which future studies into tissue- and organelle-specific V-ATPase expression, localization and function can be based.

## Introduction

The vacuolar-type H^+^-ATPase (V-ATPase) is a proton pump found in all nucleated cells of the body. Partially embedded within the membrane, its function is to transfer two hydrogen ions out of the cytoplasm at the expense of 1 ATP molecule [1]. It is thereby able to establish a proton gradient within the lumen of organelles such as lysosomes, endosomes and the trans-Golgi network. Organelle acidification is important for a diverse array of functions including intracellular trafficking and protein degradation [2]–[4].

The V-ATPase is also functionally important at the plasma membrane of specialized cell types in certain tissues. There, it is responsible for critical homeostatic functions such as body acid-base regulation (renal proximal tubule and collecting duct intercalated cells), bone remodeling (osteoclasts), and sperm storage and maturation (clear cells in the epididymis) [2], [5], [6] as well as other potential functions in other organs.

Loss of V-ATPase activity due to subunit mutations in specific cell types has been implicated in diverse pathophysiological states such as kidney and bone disease, sensorineural deafness and wrinkly skin syndrome [7]–[10]. Thus V-ATPase functions in a very broad array of physiological processes.

The V-ATPase is a large (800 kDa) and complex molecular motor. It is made up of at least 13 individual components/protein subunits organized into two functional domains: V_0_ and V_1_ [1], [4], [11], [12]. The V_0_ domain is composed of several transmembrane subunits that are involved in hydrogen ion translocation across the bilayer, while the V_1_ domain is a peripheral to the membrane and is the site of ATP hydrolysis. V_0_ is composed of 5 subunits labeled a to e while V_1_ consists of 8 subunits denoted A to H. All 13 different subunits are encoded by separate genes located throughout the genome.

Many of the 13 V-ATPase subunits exist as homologs, thereby adding another level of complexity to the motor. The diverse functions and locations of V-ATPases are believed to be encoded within the various homologs. For example the d1 subunit is ubiquitously expressed, while the d2 homolog is seen only in the kidney, osteoclast and lung [13]. Similarly two isoforms of the B subunit were initially described as so-called “kidney” (B1) and “brain” (B2) specific isoforms, although it is now clear that expression of B1 is not restricted to the kidney [12]. Currently, homologs have been identified for the a, d, e, B, C, E and G subunits [11], [14].

An additional level of V-ATPase subunit variation is encoded through splice variants. To date, splice variants have been identified for the a, d, e, C, G and H subunits [9], [13]–[23]. Just like homologs, splice variants have been shown to exhibit different expression patterns. For example two splice variants of subunit a1 are expressed in rat neurons: one variant localized to axonal varicosities while the other was sorted to distal dendrites and axons [17].

To date, discovery of V-ATPase homologs and splice variants has largely been subunit focused and experimentally based. This method has proven successful as demonstrated by the large number of identified variants. However, this fragmented discovery process led to a fragmented naming system which was recently standardized [11]. A large effort was undertaken to associate the multiple names of a given subunit to one new standard nomenclature, but extension of this nomenclature to include splice-variants was not systematically pursued at that time. Thus, here we have performed a literature search of all V-ATPase subunits, their homologs and splice variants. In addition we scanned both the RefSeq nucleotide and protein databases [24] to identify any novel subunits that have been deposited within these databases. The results of this analysis are presented here.

## Results and Discussion

At the time of the first V-ATPase nomenclature standardization, the incorporation of splice-variants was not implemented [11]. Thus, we now suggest augmenting the naming policy to allow for the differentiation of splice variants. In accordance with HUGO Gene Nomenclature Committee (HGNC) specifications, we propose the addition of a “v[1..x]” suffix to the relevant gene symbols, and “i[1..x]” to the predicted proteins. For example, ATP6V0A1v1 and ATP6V0A1v2 would differentiate between splice-variants 1 and 2 of subunit a1. As is the case at present, we propose that if new homologs of subunits with only a single currently known isoform are identified in the future, they should be named in numerical order (e. g., ATP6V1F will become ATP6V1F1 if a new isoform, which will become ATP6V1F2, is discovered). We also propose that the first full-length “known” subunit splice variant should be named “v1”, allowing for subsequent addition and standard nomenclature if additional variants are discovered in the future. For subunits with only one known variant, the v1 nomenclature should be appended to the existing name only if and when a second variant (which will be named v2) is identified. In view of our extensive database search we believe, however, that this possibility is unlikely.

**Figure 1:**
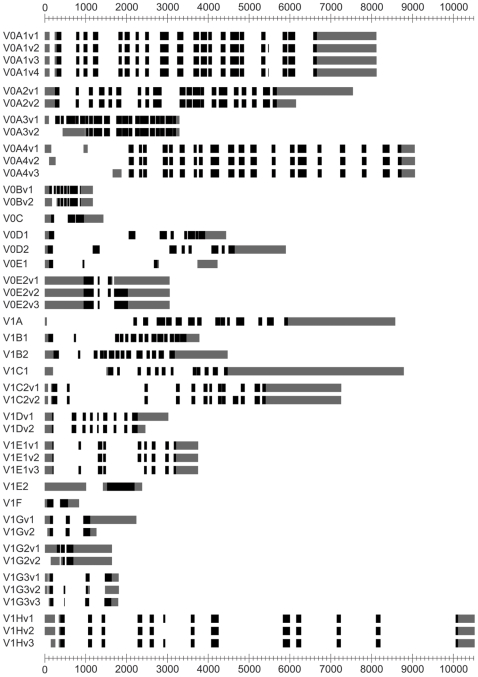
A schematic view of V-ATPase subunits. The exonic structure of all V-ATPase subunits is shown; untranslated regions are shown in light grey while protein coding regions are black. The intronic distances have been divided by 15 for display purposes. Refer to Table 1 for further information on subunits.

The V-ATPase proton pump is composed of 13 subunits. According to the data presented in Smith et al (2003) these 13 are encoded through 22 homologs. These are denoted with a “K” for known in Table 1. A simple search of the literature and the Entrez Gene database [24] reveals another 17 variants. These are denoted with either an “E” for Entrez or “L” for literature in Table 1. All of these additional subunits are splice variants except for e2, which was cloned after the publication of Smith et al (2003). Our computational analysis of sequence repositories has identified another 4 potentially novel V-ATPase variants. Again, all new sequences are encoded by splice variants; these are denoted with a “D” for database search in Table 1. It should be noted that the additional 4 variants were cloned in high-throughput mRNA discovery studies. Thus, they have no accompanying experimental validation at the protein level; instead all exist as sequenced and apparently full length mRNA transcripts. Further experimental work will be required to verify the existence of these clones in vivo; nonetheless we have included them here. Thus, with this simple literature and database search we have increased the number of V-ATPase subunit variants from 22 to 43 (Figure 1). Finally, we propose that accessory proteins (whose functions related to the V-ATPase remain unknown) that also use the ATP6 nomenclature, including ATP6AP1 and ATP6AP2, should be included in the revised nomenclature scheme, and the appropriate suffixes should be added to their names if additional isoforms and splice variants emerge in the future. However, we have also examined these sequences and apart from pseudogenes, found no variants.

**Table 1:** Overview of V-ATPase subunits and their respective RefSeq accession numbers.

	Augmented Nomenclature of Human V-ATPase Subunit Genes					
	Gene Symbol	Augmented Nomenclature	Nucleotide Accession Number	Ref	Sp	Note
	**V0 Membrane Sector**					
1	ATP6V0A1	ATP6V0A1v1	NM_001130020	[15]	B	K,E
2	--------	ATP6V0A1v2	NM_001130021	[16]	M	E,L
3	--------	ATP6V0A1v3	NM_005177	[15]	B	E,L
4#	--------	ATP6V0A1v4	DQ286422	[17]	R	L
5	ATP6V0A2	ATP6V0A2v1	NM_012463	[29]	B	K,E
6	--------	ATP6V0A2v2	BC068531	[43]	H	D
7	TCIRG1	ATP6V0A3v1	NM_006019	[18]	H	K,E
8	--------	ATP6V0A3v2	NM_006053	[19]	H	E,L
9	ATP6V0A4	ATP6V0A4v1	NM_020632	[9]	H	K,E
10	--------	ATP6V0A4v2	NM_130840	[9]	H	E,L
11	--------	ATP6V0A4v3	NM_130841	[5]	-	E
12	ATP6V0B	ATP6V0Bv1	NM_004047	[30]	H	K,E
13	--------	ATP6V0Bv2	NM_001039457	-	-	E
14	ATP6V0C	ATP6V0C*	NM_001694	[31]	B	K,E
15	ATP6V0D1	ATP6V0D1*	NM_004691	[32]	B	K,E
16	ATP6V0D2	ATP6V0D2*	NM_152565	[13]	H	K,E
17	ATP6V0E1	ATP6V0E1*	NM_003945	[33]	B	K,E
18	ATP6V0E2	ATP6V0E2v1	NM_145230	[14]	H	E,L
19	--------	ATP6V0E2v2	AK098362	-	-	D
20	--------	ATP6V0E2v3	NM_001100592	-	-	E
	**V1 Peripheral Sector**					
21	ATP6V1A	ATP6V1A*	NM_001690	[34]	B	K,E
22	ATP6V1B1	ATP6V1B1*	NM_001692	[35]	H	K,E
23	ATP6V1B2	ATP6V1B2*	NM_001693	[36]	H	K,E
24	ATP6V1C1	ATP6V1C1*	NM_001695	[37]	B	K,E
25	ATP6V1C2	ATP6V1C2v1	NM_144583	[13]	H	K,E
26	--------	ATP6V1C2v2	NM_001039362	[13]	H	L,E
27	ATP6V1D	ATP6V1Dv1*	NM_015994	[38]	M	K,E
28	--------	ATP6V1Dv2*	AF100741	[44]	H	D
29	ATP6V1E1	ATP6V1E1v1	NM_001696	[39]	B	K,E
30	--------	ATP6V1E1v2	NM_001039366	-	-	E
31	--------	ATP6V1E1v3	NM_001039367	-	-	E
32	ATP6V1E2	ATP6V1E2*	NM_080653	[40]	H	K,E
33	ATP6V1F	ATP6V1F*	NM_004231	[41]	H	K,E
34	ATP6V1G1	ATP6V1G1v1	NM_004888	[42]	B	K,E
35	--------	ATP6V1G1v2	BC008452	[43]	H,M	D
36	ATP6V1G2	ATP6V1G2v1	NM_130463	[21]	H	K,E
37	--------	ATP6V1G2v2	NM_138282	[22]	R,B,C	L,E
38	ATP6V1G3	ATP6V1G3v1	NM_133262	[13]	H	K,E
39	--------	ATP6V1G3v2	NM_133326	-	-	E
40	--------	ATP6V1G3v3	BC101129	[13]	H	L
41	ATP6V1H	ATP6V1Hv1	NM_015941	[23]	B	K,E
42	--------	ATP6V1Hv2	NM_213619	[23]	B	L,E
43	--------	ATP6V1Hv3	NM_213620	-	-	E

A list of the current HUGO Gene Nomenclature Committee (HGNC) designations for the known V-ATPase subunits is provided, along with the corresponding subunits described in this report. An augmented naming system (Proposed) is implemented to incorporate the multiple splice variants identified in this analysis.

Notes for Table 1 .

Ref - Original cloning paper.

Sp - Species gene was originally cloned from:

B - Bovine; M - Mouse; R - Rat; H - Human; C- Chicken.

K - known variant noted in Smith AN et al (2003).

E - entrez gene entry exists.

L - identified in literature search.

D - cloned in a high-throughput experiment, needs further experimental validation.

#No human transcript could be identified in RefSeq, so the rat ortholog is provided.

*If a novel homolog is discovered for a subunit with no known homologs then the current homolog will be denoted −1 and the novel homolog denoted −2. For example ATP6V1F will become ATP6V1F1 and the novel homolog ATP6V1F2. If additional splice variants of these new homologs are then discovered, the terminology will become, for example, ATP6V1F1v1 and ATP6V1F1v2 etc.

Experimentally identifying and characterizing the associated functional proteins was outside the scope of this study but should be the subject of future work by investigators interested in V-ATPase function, including our own groups. The results of this study suggest that the V-ATPase is regulated in a much more complex manner than is currently assumed. At what level this regulation is exerted remains to be determined experimentally, and having a complete systems overview of all V-ATPase components will help expedite this process. This almost two-fold increase in the number of V-ATPase subunit forms demonstrates how the output of broad genomic scale projects can be utilized for a specialized pursuit. It also highlights the importance of computational methods for sifting and sorting through vast amounts of data deposited in sequence databases worldwide. Identification and standardization of transcript variation offers a powerful approach to guide the future assignation of functional significance among protein variants.

## Materials and Methods

Sequences corresponding to the 13 human V-ATPase subunits described previously [11] were retrieved from the HUGO Gene Nomenclature Database (HGNC) database [25]. These sequences were used to query the RefSeq non-redundant protein and nucleotide databases [24] with the BLAST algorithm [26]. Various modules from the Bioperl toolkit were utilized to process the resulting BLAST output [27]. The HGNC V-ATPase identifiers were used to query the SpliceCenter databases [28]. The results of this analysis were manually integrated with the BLAST results described above. This integrated catalogue is presented in Table 1 and Figure 1.
